# Migration Intent of Health Care Workers during the COVID-19 Pandemic in Kosovo

**DOI:** 10.3390/ijerph191711122

**Published:** 2022-09-05

**Authors:** Nora Murataj, Blerim Syla, Yllka Krasniqi, Shegë Bahtiri, Dardan Bekaj, Petrit Beqiri, Ilir S. Hoxha

**Affiliations:** 1 Federata e Sindikatave të Shëndetësisë së Kosovës, 10000 Prishtina, Kosovo; 2 Institute of South East Europe for Health and Social Policy, 10000 Prishtina, Kosovo; 3 Advanced Nursing Practices Department, Heimerer College, 10000 Prishtina, Kosovo; 4 Research Unit, Heimerer College, 10000 Prishtina, Kosovo; 5 Evidence Synthesis Group, 10000 Prishtina, Kosovo; 6 The Dartmouth Institute for Health Policy and Clinical Practice, Geisel School of Medicine at Dartmouth, Lebanon, NH 03766, USA

**Keywords:** COVID-19, migration, healthcare workers, public health

## Abstract

The migration of healthcare workers from developing countries to more economically developed countries is a long-standing and ongoing trend. Loss of qualified staff due to migration can negatively impact healthcare systems. Understanding factors that drive migration is essential to identifying and managing health system needs. Our study explored factors related to the migration intent of healthcare staff in Kosovo, particularly after the COVID-19 pandemic. We carried out a cross-sectional survey of healthcare workers from public and private institutions. The survey analysed the prevalence of willingness to migrate and whether willingness was affected by the pandemic, and calculated crude and adjusted odds ratios for variables which may influence migration willingness. 14.43% of healthcare workers reported aspiration to migrate, and 23.68% reported an increased chance of migrating after the pandemic. Dissatisfaction with wages and working conditions, higher education and private sector engagement were associated with increased odds of migration willingness. After the pandemic, factors related to interpersonal relationships and state response gave lower odds of migration intent. These findings point to potential factors associated with the migration of healthcare workers, which can help policymakers address gaps in national health system strategy.

## 1. Introduction

The migration of healthcare workers (HCWs) to more economically developed countries, driven by global HCW shortages and high demand, is likely to continue in the coming years. Over a quarter of physicians and 16% of nurses in OECD countries are foreign-born [1]. HCW migration trends contribute to the loss of qualified health personnel from developing countries, impacting their capacity to deliver equitable and quality health care [2].

Lee’s ‘push–pull’ theory of migration [3] examines the migration of highly skilled workers, which includes HCWs. In the healthcare context, pull factors refer to factors that drive potential migrants towards destination countries, such as better pay, more professional and social opportunities, and improved quality of life [4,5].

Factors that ‘push’ HCWs from countries of origin include poor working conditions, political environment, and cultural factors. In Lithuania, Gostautait et al. [6] found that financial dissatisfaction is a strong predictor for migration intentions of nurses and residents. Remuneration interacts with additional working and other characteristics, including education level, work conditions, and burnout, in their decision to migrate [7].

Other research has shown the complexity of push factors in migration. Anastasiou [8] found that Greek physicians reported the cultural mindset of their country as an influential factor in the decision to migrate, and political insecurity was identified as a pertinent factor in migration from South Africa by Labonte et al. [9].

The concept of ‘brain drain’, which indicates the excessive shortage of qualified workforce as a result of migration [10], follows the push-pull model. The brain drain of HCWs produces an array of effects. For countries in the developing and lower income categories, the shortage of physicians affects the quality of health services and healthcare coverage, particularly in rural areas [11,12]. In addition, nursing shortages have been associated with more errors and undermining of patient safety [13].

These factors have also been observed in studies of HCW migration from the Western Balkans [14]. In Serbia, for example, Vracic [15] reported that over 800 certificates allowing doctors to work abroad are issued per year. HCW migrants from the Western Balkans have also previously cited wage dissatisfaction, professional development, and political instability at home, as reasons for migration [16].

In Kosovo, the migration of healthcare staff to EU countries, including physicians, nurses, and technical staff, is considered detrimental to the system [17]. Reports have shown that up to 60% of nurses have expressed willingness to migrate, which was attributed to higher salaries and opportunities for professional development abroad, and a lack of suitable available positions in Kosovo [18].

Some target destination countries for Kosovo HCWs rely on the migrant healthcare workforce, with Switzerland counting over 30% of its health workforce as foreign-born [19]. During the COVID-19 pandemic, many OECD countries, which were already reliant on migrant health personnel, advanced procedures to recognise the qualifications of foreign HCWs to aid in meeting the added demands of the crisis [19].

Some studies have looked at trends and factors related to healthcare staff migration during the pandemic [9,20,21], and this study further expands this perspective by examinings the trends in Kosovo. Data and tracking of migrant characteristics are necessary for creating retention policies and better matching between health labour supply and education investments [22].

This study investigated the willingness of HCWs in Kosovo to migrate, focusing on potential influencing variables. In addition, the study aims to assess how intent to migrate has been affected by the COVID-19 pandemic in relation to other relevant health system variables.

### 1.1. Background

#### 1.1.1. COVID-19 in Kosovo

Kosovo registered its first COVID-19 cases in March 2020, which was closely followed by the implementation of a national lockdown [23]. The government issued emergency financial support packages, and only businesses considered essential were allowed to operate [23]. Restrictions were lifted according to waves of infection. They were reintroduced in response to the wave of winter 2020, and included curfews, work-from-home mandates, restrictions on travel, and gastronomy and entertainment industries [23].

In January 2021, the government passed an economic recovery package. Healthcare staff engaged in the frontlines of the COVID-19 response received support through additional salaries. Kosovo launched its vaccination campaign in March 2021, with restrictions relaxed [23].

#### 1.1.2. Kosovo Health System

Kosovo’s national health care system is mainly subsidised by the government through taxes [24]. However, private institutions have a significant share in service provision. Reports from 2014 [25] show that Kosovo citizens spend around 33% out-of-pocket on healthcare, mainly on pharmaceuticals and medical devices. There are initiatives to reform and enhance the primary sector, which is reportedly underutilized [26]. Kosovo has committed to a universal healthcare insurance program [27], but its implementation is yet to begin.

The only higher education institutions training medical doctors are public universities [28], whereas other public and private universities offer other professional healthcare programs.

## 2. Materials and Methods

### 2.1. Design and Instruments

We employed a cross-sectional design in our study, using previously validated instruments for survey preparation [29,30]. The questionnaire was divided into three sections. The first part included demographic questions. The second part consisted of questions related to factors influencing migration, including sector (private vs. public), level of healthcare institution (primary, secondary and tertiary), and satisfaction with working conditions and wages. In this part, we asked the first main outcome question ‘Would you migrate’, indicating a general preference for migration. The final part consisted of questions relating to experiences during the COVID-19 pandemic, including interpersonal relationships, emotional state, working conditions and workload, receipt of supplementary payments, and government relief measures during the pandemic, also referred to as government attitude. In this part, we also asked the second main outcome question ‘Have your chances of migrating after the pandemic increased?’, indicating the preference to migrate after the pandemic situation has subsided.

### 2.2. Participants and Procedure

We obtained the study sample, using the member list of the Trade Union Health Federation of Kosova. The Trade Union Health Federation of Kosova is a representative organization of health workers in the Republic of Kosovo. It consists of 51 trade unions and union sections organized through health institutions in the entire territory of the Republic of Kosovo [31]. It has over 11,000 members, including medical and health professionals, administrative staff employed in medical institutions, and other non-medical support workers [31]. We used systematic random sampling, selecting every fifth member of the list for a total of 1622 participants. Administrative and other non-medical staff were excluded. The list includes physicians, nurses and other healthcare workers such as lab technicians and medical support teams.

Initially, the survey was piloted and revised based on feedback from HCWs. Interviewers were trained one month prior to data collection. The interviewers then administered telephone surveys to the final list of participants between December 2020 and January 2021. They read a standard information sheet related to the study, which explained conditions of confidentiality and the study’s purpose. In addition, they informed participants that in case of discomfort or personal reasons they may interrupt the survey at any time or retract information. Each participant was assigned a code to ensure confidentiality and anonymity upon data entry. The overall response rate was 65.5%, giving a final sample of 1061 participants. Each participant consented to participate in the study before the interview was administered. An ethical permit was obtained from Heimerer College in Prishtina.

### 2.3. Data Analysis

In the first step, we calculated the prevalence of willingness to migrate in our sample using survey responses to the general question ‘Would you migrate’ and ‘Have your chances of migrating after the pandemic increased?’.

We performed univariable logistic regression to analyse associations of several demographic, system and individual variables. The main outcomes variables, then, were odds for migration willingness in general, and migration willingness after the pandemic. Variables with a *p*-value < 0.10 from the unadjusted univariable analyses were used in two final multiple logistic regression models. Variance Inflation Factor (VIF) analysis of the models showed no collinearity between variables. Data analysis was performed with STATA, release V.17 BE (StataCorp, Lakeway, TX, USA).

## 3. Results

Table 1 shows demographic information on the participants. Table 2 shows summarized responses of participants’ preference for migration. 14.43% of respondents stated willingness to migrate. 20.81% of physicians. In addition, 23.68% reported increased chances of migrating after the pandemic. A important part of nurses (24.01%) and other medical professionals (32.61%) prefer to migrate.

### 3.1. Migration Intent

#### 3.1.1. Demographic Factors

Results from the regression model (Table 3) suggest that increased age is associated with a small but significant decrease in the odds of migration intent (OR = 0.96, 95% CI [0.94–0.97], *p* < 0.001), and that professionals with higher education were more likely to favour migration (aOR = 2.15, 95% CI [1.47–3.13], *p* < 0.001). In addition, physicians had higher odds of migration intent compared to nurses and other professionals (OR = 1.79, 95% CI [1.19–2.70], *p* < 0.01). Gender and residence of HCWs were not associated with significant differences in the odds for migration.

#### 3.1.2. System Factors

Professionals from primary care centres were less likely to favour migration compared to those from the University Clinical Centre (tertiary level of care) and regional hospitals (aOR = 0.45, 95% CI [0.29–0.70], *p* < 0.001). Respondents working in the private sector were more likely to prefer migration (OR = 1.68, 95% CI [1.01–2.79], *p* < 0.05), compared with those working in the public sector. For the working conditions variable, respondents who were dissatisfied were more likely to prefer migration compared to those who were neutral or satisfied (aOR = 2.29, CI [1.49–3.52], *p* < 0.001). Professionals who responded neutral (aOR = 3.83, CI [1.24–11.9], *p* < 0.05) or dissatisfied (aOR = 1.84, CI [1.22–2.76], *p* < 0.01) in terms of wage satisfaction were also more likely to prefer migration.

### 3.2. Migration after the Pandemic

#### 3.2.1. Demographic Factors

Table 4 shows regression model results for migration intent after the pandemic. Of demographic variables, only gender led to a difference in odds, with men less likely to favour migration compared to women (aOR = 0.66, 95% CI (0.47–0.93), *p* < 0.05).

#### 3.2.2. System Factors

Compared to nurses, physicians were less likely to report increased chances of migration (OR = 0.52, 95% CI [0.34–0.81], *p* < 0.01), whereas other medical professionals were more likely (OR = 1.53, 95% CI [1.07–2.18], *p* < 0.01). Respondents from primary care centres had higher odds of reporting increased chances for migration post-pandemic (OR= 3.63, 95% CI [2.39–5.51], *p* < 0.001). There was no significant difference in odds between institutional sectors. Most participants agreed that work conditions worsened during the pandemic, the workload increased, and they received pay supplements, but these variables were not significantly associated with migration willingness post-pandemic. Moreover, satisfaction with wages and conditions did not significantly predict the odds of migrating after the pandemic.

#### 3.2.3. Individual and Social Factors

Participants who responded neutral to whether interpersonal relations improved during the pandemic had lower odds for migration preference post-pandemic compared to other respondents (OR = 0.49, 95% CI [0.31–0.76], *p* < 0.01). Most respondents agreed that the pandemic had influenced their emotional state, but this factor was not significantly associated with migration preference post-pandemic.

Finally, participants who did not report an effect of the government’s attitude towards the pandemic on their intent to migrate had lower odds of intent to migrate post-pandemic (Neutral, aOR = 0.35, 95% CI [0.21–0.57], *p* < 0.001, Disagree, aOR = 0.49, 95%CI [0.30–0.68] *p* < 0.001).

## 4. Discussion

Our study explored health care worker migration intent in Kosovo, including the effect of the COVID-19 pandemic on migration intent. We found that 14.43% of respondents were willing to migrate, and that almost a quarter report increased likelihood of migration after the pandemic.

Dissatisfaction with wages and working conditions, working as a physician, and being engaged in the private sector were factors that increased the likelihood of willingness to migrate.

Our findings are in agreement with those of previous studies, which have reported that dissatisfaction with wages and working conditions are strongly associated with migration preference among health workers [32,33,34]. Bidwell [35] reported that better working conditions are a crucial factor in retaining physicians in countries of origin.

Physicians were generally more likely to report intention to migrate than nurses and other professionals. A recent migrant profiling study by Adeniyi et al. [36] reported that early-career physicians migrate to seek better postgraduate training and higher salaries. In general, healthcare professionals from developing countries migrate to countries with robust health systems and more opportunities for advancement [22,37].

Our study suggests that the willingness of private sector employees to migrate may also be linked to working conditions. This finding is coherent with overall migration trends in Kosovo, which indicate that private sector employees in the country are more inclined to migrate [17]. Motives for migration may also be related to wages. Private sector salaries in Kosovo can be lower than public enterprises [38], a factor previously reported as a motive for healthcare staff to migrate [39]. Professionals often mitigate dissatisfaction with salary or conditions by engaging in a dual practice in both private and public sectors [40,41].

Professionals from regional hospitals (secondary) and primary care were less likely to favour migration than those in the University Clinical Centre, a tertiary public health institution. Similarly, higher education was a strong predictor of migration willingness. Though our study did not explore professional motivations, migrant physicians with more highly specialised profiles are reported to have more well-defined career orientation and thus favour migration due to the lack of professional opportunities in their country of origin [29,36,42]. Similarly, Humphries et al.’s [43] typology assessment of doctors showed that career-oriented physicians were the most common type (60%) of non-EU migrant doctors in Ireland. A country case study by Apostu et al. [44] in Romania also found that professionals with highly specialised skills favour migration, as they can use their skills more effectively in developed countries.

We also examined whether willingness to migrate has been affected by the COVID-19 pandemic. Nearly a quarter of respondents considered their migration preference to have increased after the pandemic, with physicians less likely to report a change compared to nurses and other medical staff.

Interestingly, HCWs from the primary care sector were more likely to favour migration after the pandemic. Humphries et al. [20] found that HCWs had observed some improvement in working conditions during the pandemic due to added support in terms of personnel and funding. In parallel, during the pandemic response in Kosovo, the leading University clinic and regional hospitals saw more additional staffing and support compared to primary care. This disparity may have influenced the perceptions of HCWs at the primary care level.

Salary supplements did not significantly predict the odds of migration intent. Most respondents received pay supplements during the pandemic and reported higher wage satisfaction for this period. Previously, supplementing salary alone was reported as insufficient to prevent physician migration. For example, Apostu et al. [44] reported that salary additions without other active measures had a low impact on migrant physician retention in Romania. In our study, a minimal number of HCWs did not receive supplements, which affects the chances of observing any differences.

Men were less likely to report increased migration preference after the pandemic than women. Apostu and Vasile [45] have previously reported that female physicians from Romania were more likely to migrate due to several factors, but mainly due to their wider range of applicable specialities.

We found that some interpersonal variables were related to changes in the odds of post-pandemic migration. HCWs who perceived that the pandemic did not affect their interpersonal relationships and who were neutral towards the governmental response had lower odds of increased migration intent after the pandemic. These findings relate to those of a study by Adeniyi et al. [36] before the pandemic, which reported that political factors had a low influence (5.5%) on Nigerian HCWs. Instead, they reported that family ties and the desire to serve the nation were the main motives to stay in their home countries. Social factors were also examined in HCW migration during the pandemic. According to Humphries et al. [20], physicians also considered their relationship to home and country of origin as influential in their decision to remain and work in Ireland during the pandemic.

Our findings point to specific risks for Kosovo due to HCW migration. Earlier reports show already low ratios of physicians and nurses to the population, compared to the region and the EU [46]. This also applies for general practitioners and primary health care physicians, for which the need and difficulties to recruit have already been reported [26].

Findings also point to the economic costs of HCW migration. Some estimates indicate around 100,000 EUR are spent annually to educate one physician [47]. Beyond training and educational costs, according to the model by Saluja et al. [48], which also assesses the Eastern Mediterranean region, lower and middle income countries lose nearly 16 billion annually due to under-five mortality that results from loss of physicians due to migration.

## 5. Conclusions

This study explored factors related to the willingness of healthcare professionals in Kosovo to migrate, both generally and after the pandemic. Dissatisfaction with wages and conditions, engagement in the private sector, and physicians compared with other HCWs, were associated with increased intent to migrate.

After the pandemic, almost a quarter of the professionals reported increased intention to migrate. Male HCWs, professionals who felt their personal lives were unaffected by the pandemic, and those who were neutral to the government’s actions, were also less inclined to perceive the pandemic as a ’push’ factor for migrating.

This study was the first to explore the role of the pandemic in the migration of healthcare professionals in Kosovo. Previously established ‘push’ factors for migration were examined through the questionnaire, which was expanded to include additional and pandemic-related factors. The survey was compiled in collaboration with public health experts and was piloted before launch.

### 5.1. Limitations

The study’s main limitation was the use of a cross-sectional design, with data collected at one-time point in the pandemic.

In addition, we conducted a phone survey based on the member information of the Trade Union Federation of Health in Kosovo. Members consent to providing their contact information for relevant studies. Although phone surveys provide good geographical coverage and enable quicker data collection, they are susceptible to non-response bias compared to other surveying methods. We obtained a response rate within the range of other similar studies [48,49]. However, to ensure reduction of bias, future studies should employ other survey approaches, and consider using a mixed methods approach. Employing a qualitative method to support quantitative findings could provide extensive information beyond what is captured by this survey design.

### 5.2. Policy Implications

Various short, medium and long term measures have been implemented to address HCW migration. A recent review [50] noted that HCW migration flows are impacted by factors other than professional, macroeconomic and political, including family and social networks, which are more difficult to address. Long-term policy action should target workforce sustainability, which includes personnel development and health labour market measures.

For example, Ecuador has addressed its lack of training opportunities through a scholarship program which finances education abroad with compulsory return, achieving a very high return rate [51]. Kosovo has previously applied similar programs, but not for medical studies. Ifanti et al. [52] recommended a policy approach that addresses job insecurity as a push factor for young medical graduates, through the implementation of programmes that directly link the labour market with recent graduates to improve access to jobs.

Other measures for mitigating the effects of HCW migration include agreements between countries which enable brain circulation, i.e., the return of skills and knowledge including through exchange or training opportunities [53]. In this regard, according to Radwan and Sakr [54], mobility platforms between Africa and the EU have proven beneficial in creating appropriate infrastructure, better payment conditions and career advancement programmes for highly qualified academics. However, according to Dhillon et al. [55], developing countries may have less capacity and potential to influence bilateral agreements, which presents additional challenges for managing migration.

Migration of South-eastern European HCWs to other European countries is a worrying trend. Due to the ageing of the health workforce in more developed countries, demand for migrant HCWs is expected to continue [20]. Countries of origin should track and monitor the migration of HCWs, particularly those who are highly specialised, to initiate response strategies. To prevent personnel loss in different areas, policymakers should also consider internal policies that address gaps in working conditions, advancement opportunities, and remuneration.

Our findings provide vital information for policy, considering the lack of data specifically related to the migration of the healthcare workforce in Kosovo. Follow-up studies can benefit from time-series designs, enabling the tracking of trends and migrant profiles across systemic variables. Future research can expand on additional phases of the migration cycle, including factors for return and circular migration.

## Figures and Tables

**Table 1:** Characteristics of study participants.

	n	%
Physician	197	18.58%
Nurse	679	64.06%
Other	184	17.36%
Age	49.86 *	
Gender (Male)	320	30.19%
Higher education	450	42.45%
Experience	24.12 *	

* Mean.

**Table 2:** Summary results of migration intent among healthcare workers.

	Yes	Total	%
**Are you willing to migrate?**	153	1060	14.43%
Physician	41	197	20.81%
Nurse	87	679	12.81%
Other	25	184	13.59%
**Have your chances of migrating after the pandemic increased?**	251	1060	23.68%
Physician	28	197	14.21%
Nurse	163	679	24.01%
Other	60	184	32.61%

**Table 3:** Preference for migration.

	Yes	No	Crude Odds Ratio (95%CI)		*p*-Value	Adjusted Odds Ratio (95%CI)		*p*-Value
	Events/Total (%)	Events/Total (%)						
**Age (years)**	49.86 *	9.52 **	0.96 (0.94–0.97)		<0.001			
**Gender (Male)**	45/153 (29.4)	275/907 (30.3)	0.96 (0.66–1.39)		0.821			
**Urban**	123/153 (80.4)	731/907 (80.6)	0.99 (0.64–1.52)		0.953			
**Higher education**	95/153 (62.1)	355/907 (39.1)	2.55 (1.79–3.62)		<0.001	2.15 (1.47–3.13)		<0.001
**Type of healthcare worker**								
Nurse	87/153 (56.9)	592/907 (65.3)	Reference					
Physician	41/153 (26.8)	156/907 (17.2)	1.79 (1.19–2.70)		0.006			
Other	25/153 (16.3)	159/907 (17.5)	1.07 (0.66–1.73)		0.782			
**Experience (years)**	24.12 *	10.26 **	0.98 (0.96–1.00)		0.016			
**Institution**								
University clinical centre	58/153 (37.9)	198/907 (21.8)	Reference			Reference		
Primary care centres	49/153 (32.0)	448/907 (49.4)	0.37 (0.25–0.57)		<0.001	0.45 (0.29–0.70)		<0.001
Regional hospitals	46/153 (30.1)	261/907 (28.8)	0.60 (0.39–0.92)		0.020	0.75 (0.47–1.18)		0.211
**Working in the private sector**								
No	128/153 (83.7)	802/907 (88.4)	Reference					
Yes	22/153 (14.4)	82/907 (9.0)	1.68 (1.01–2.79)		0.044			
Refuses to answer	3/153 (2.0)	23/907 (2.5)	0.82 (0.24–2.76)		0.745			
**Satisfaction with working conditions**								
Satisfied	103/153 (67.3)	779/907 (85.9)	Reference			Reference		
Neutral	2/153 (1.3)	15/907 (1.7)	1.01 (0.23–4.47)		0.991	0.76 (0.15–3.92)		0.747
Dissatisfied	48/153 (31.4)	113/907 (12.5)	3.21 (2.16–4.77)		<0.001	2.29 (1.49–3.52)		<0.001
**Satisfaction with wage**								
Satisfied	42/153 (27.5)	430/907 (47.4)	Reference			Reference		
Neutral	5/153 (3.3)	18/907 (2.0)	2.84 (1.00–8.05)		0.049	3.83 (1.24–11.9)		0.020
Dissatisfied	106/153 (69.3)	459/907 (50.6)	2.36 (1.62–3.46)		<0.001	1.84 (1.22–2.76)		0.004

* Mean, ** Standard deviation.

**Table 4:** Preference for migration post-pandemic.

	Yes	No	Crude Odds Ratio (95%CI)	*p*-Value	Adjusted Odds Ratio (95%CI)	*p*-Value
	Events/Total (%)	Events/Total (%)				
**Age (years)**	49.86 *	9.52 **	0.99 (0.98–1.00)	0.171		
**Gender (Male)**	62/251 (24.7)	258/809 (31.9)	0.70 (0.51–0.97)	0.031	0.66 (0.47–0.93)	0.018
**Urban**	200/251 (79.7)	654/809 (80.8)	0.93 (0.65–1.32)	0.685		
**Higher education**	116/251 (46.2)	334/809 (41.3)	1.22 (0.92–1.62)	0.168		
**Type of healthcare worker**						
Nurse	163/251 (64.9)	516/809 (63.8)	Reference			
Physician	28/251 (11.2)	169/809 (20.9)	0.52 (0.34–0.81)	0.004		
Other	60/251 (23.9)	124/809 (15.3)	1.53 (1.07–2.18)	0.019		
**Experience (years)**	24.12 *	10.26 **	0.99 (0.97–1.00)	0.041	0.98 (0.97–1.00)	0.020
**Institution**						
University clinical centre	33/251 (13.1)	223/809 (27.6)	Reference		Reference	
Primary care centres	167/251 (66.5)	330/809(40.8)	3.42 (2.27–5.15)	<0.001	3.63 (2.39–5.51)	<0.001
Regional hospitals	51/251 (20.3)	256/809 (31.6)	1.35 (0.84–2.16)	0.218	1.31 (0.81–2.14)	0.272
**Working in the private sector**						
No	225/251 (89.6)	705/809 (87.1)	Reference			
Yes	17/251 (6.8)	87/809 (10.8)	0.61 (0.36–1.05)	0.075		
Refuses to answer	9/251 (3.6)	17/809 (2.1)	1.66 (0.73–3.77)	0.227		
**Satisfaction with working conditions**						
Satisfied	209/251 (83.3)	673/809 (83.2)	Reference			
Neutral	2/251 (0.8)	15/809 (1.9)	0.43 (0.10–1.89)	0.264		
Dissatisfied	40/251 (15.9)	121/809 (15.0)	1.06 (0.72–1.57)	0.753		
**Satisfaction with wage**						
Satisfied	110/251 (43.8)	362/809 (44.7)	Reference			
Neutral	6/251 (2.4)	17/809 (2.1)	1.16 (0.45–3.02)	0.759		
Dissatisfied	135/251 (53.8)	430/809 (53.2)	1.03 (0.77–1.38)	0.824		
**Interpersonal relations have improved during the pandemic**						
Agree	139/251 (55.4)	442/809 (52.2)	Reference			
Neutral	27/251 (10.8)	168/809 (20.8)	0.49 (0.31–0.76)	0.002		
Disagree	85/251 (33.9)	219/809 (27.1)	1.18 (0.86–1.62)	0.308		
**The working conditions have worsened during the pandemic**						
Agree	165/251 (65.7)	553/809 (68.4)	Reference			
Neutral	14/251 (5.6)	38/809 (4.70)	1.23 (0.65–2.33)	0.516		
Disagree	72/251 (28.7)	218/809 (26.9)	1.11 (0.81–1.52)	0.531		
**The pandemic has influenced my emotional state**						
Agree	233/251 (92.8)	739/809 (91.3)	Reference			
Neutral	2/251 (0.8)	9/809 (1.1)	0.70 (0.15–3.29)	0.656		
Disagree	16/251 (6.4)	61/809 (7.5)	0.83 (0.47–1.47)	0.527		
**Received supplementary payments during the pandemic**						
Agree	242/251 (96.4)	794/809 (98.1)	Reference			
Neutral	1/251 (0.4)	2/809 (0.2)	1.64 (0.15–18.20)	0.687		
Disagree	8/251 (3.2)	13/809 (1.6)	2.02 (0.83–4.93)	0.123		
**Pandemics has increased the work overload**						
Agree	229/251 (91.2)	715/809 (88.4)	Reference			
Neutral	7/251 (2.8)	13/809 (1.6)	1.68 (0.66–4.26)	0.274		
Disagree	15/251 (6.0)	81/809 (10.0)	0.58 (0.33–1.02)	0.060		
**Government institution’s attitude has influenced my decision to migrate**						
Agree	45/251 (17.9)	68/809 (8.4)	Reference			
Neutral	49/251 (19.5)	212/809 (26.2)	0.35 (0.21–0.57)	<0.001		
Disagree	157/251 (62.5)	529/809 (65.4)	0.49 (0.30–0.68)	<0.001		

* Mean, ** Standard Deviation.

## Data Availability

The datasets used and/or analysed during the current study are available from the corresponding author on reasonable request.
